# A Genome-Wide Association Study for Tolerance to Paratuberculosis Identifies Candidate Genes Involved in DNA Packaging, DNA Damage Repair, Innate Immunity, and Pathogen Persistence

**DOI:** 10.3389/fimmu.2022.820965

**Published:** 2022-04-06

**Authors:** María Canive, Gerard Badia-Bringué, Patricia Vázquez, Joseba M. Garrido, Ramón A. Juste, Almudena Fernandez, Oscar González-Recio, Marta Alonso-Hearn

**Affiliations:** ^1^ Department of Animal Health, NEIKER-Basque Institute for Agricultural Research and Development, Basque Research and Technology Alliance (BRTA), Derio, Spain; ^2^ Departamento de Mejora Genética Animal, Instituto Nacional de Investigación y Tecnología Agraria y Alimentaria, CSIC, Madrid, Spain; ^3^ Departamento de Producción Agraria, Escuela Técnica Superior de Ingeniería Agronómica, Alimentaria y de Biosistemas, Universidad Politécnica de Madrid, Ciudad Universitaria, Madrid, Spain

**Keywords:** disease tolerance, paratuberculosis, DNA packaging, DNA damage repair, innate immunity, tissue damage control, pathogen persistence

## Abstract

Although the genetic susceptibility to diseases has been extensively studied, the genetic *loci* and the primary molecular and cellular mechanisms that control disease tolerance are still largely unknown. Bovine paratuberculosis (PTB) is an enteritis caused by *Mycobacterium avium* subsp. *paratuberculosis* (MAP). PTB affects cattle worldwide and represents a major issue on animal health. In this study, the associations between host genetic and PTB tolerance were investigated using the genotypes from 277 Spanish Holstein cows with two distinct phenotypes: cases) infected animals with positive PCR and bacteriological culture results but without lesions in gut tissues (N= 24), and controls) animals with negative PCR and culture results but with PTB-associated lesions (N= 253). DNA from peripheral blood of the study population was genotyped with the Bovine EuroG MD Bead Chip, and the corresponding genotypes were imputed to whole-genome sequencing (WGS) data. A genome-wide association study was performed using the WGS data and the defined phenotypes in a case-control approach. A total of 142 single nucleotide polymorphisms (SNPs) were associated (false discovery rate ≤ 0.05, *P* values between 1.5 × 10^-7^ and 5.7 × 10^-7^) with tolerance (heritability= 0.55). The 40 SNPs with *P*-values < 5 × 10^-7^ defined 9 QTLs and 98 candidate genes located on BTA4, BTA9, BTA16, BTA25, and BTA26. Some of the QTLs identified in this study overlap with QTLs previously associated with PTB, bovine tuberculosis, mastitis, somatic cell score, bovine diarrhea virus persistent infection, tick resistance, and length of productive life. Two candidate genes with important roles in DNA damage response (*ERCC4* and *RMI2)* were identified on BTA25. Functional analysis using the 98 candidate genes revealed a significant enrichment of the DNA packaging process (*TNP2/PRMI1/PRM2/PRM3*). In addition, the *TNF*-signaling (bta04668; *TRAF5/CREB5/CASP7/CHUK*) and the toxoplasmosis (bta05145; *TGFβ2/CHUK/CIITA/SOCS1*) pathways were significantly enriched. Interestingly, the *nuclear Factor NF-κβ Inhibitor Kinase Alpha* (*CHUK*), a key molecule in the regulation of the *NF-κB* pathway, was enriched in both pathways. Taken together, our results define a distinct immunogenetic profile in the PTB-tolerant animals designed to control bacterial growth, modulate inflammation, limit tissue damage and increase repair, thus reducing the severity of the disease.

## Introduction

Paratuberculosis (PTB) or Johne´s disease is a granulomatous enteritis of domestic and wild ruminants caused by *Mycobacterium avium* subsp. *paratuberculosis* (MAP). PTB is a major problem for animal health and must be reported to the World Organization for Animal Health. In Europe and North America, PTB is endemic in dairy cattle, with herd prevalence estimates higher than 50% (1). PTB causes substantial economic losses to the dairy industry mainly due to decreased milk production, weight loss, and premature culling or death of the infected animals (2). Animals are infected early in life through the fecal-oral route but clinical onset appears when animals are 18 months or older. Current diagnostic tests, ELISA and fecal PCR, often fail to detect MAP-infected cattle early in the course of infection (3, 4). According to their extension, cellular infiltrate, and amount of MAP, PTB-associated histological lesions have been classified into the following categories: focal, multifocal, and diffuse (diffuse paucibacillary, diffuse intermediate, and diffuse multibacillary) (5). High bacterial burden, clinical signs, and gross lesions are associated with the presence of diffuse lesions. MAP has been associated with Crohn´s disease (CD) in humans and has been detected in samples of patients with CD, ulcerative colitis, and idiopathic inflammatory bowel disease (IBD)-associated colorectal cancer (6–8). MAP is also a possible trigger factor in some human autoimmune diseases such as rheumatoid arthritis, multiple sclerosis, and type I diabetes (9–12). Currently, no efficacious vaccine exists that confers protection against MAP. The current control measures include the removal of MAP-infected animals from the herd and the enhancement of farm-biosecurity measures. Genetic selection to enhance the resistance of dairy cattle to PTB and other diseases is extensively being explored (13, 14).

In previous studies, we identified a total of 380 single nucleotide polymorphisms (SNPs) associated (FDR ≤ 0.05, *P* < 5 × 10^-7^) with PTB susceptibility using serum ELISA for the detection of humoral responses against MAP and post-mortem diagnostic definitions (tissue PCR, tissue bacteriological culture, and histopathological analysis) in a common set of Spanish Holstein cattle (N= 983) (15, 16). These studies showed that PTB susceptibility is under complex genetic control, with heritability (h^2^) estimates ranging from 0.139 for the combination of ELISA-PCR-culture positive results to 0.189 for the risk of developing diffuse lesions. Using these phenotypes, we were, however, unable to identify SNPs associated with PTB resistance. While test positive cows are very likely infected, animals can test negative due to the lack of sensitivity of the diagnostic method or lack of exposure to MAP. Host defense strategies against infectious diseases are comprised of both host resistance and disease tolerance. Resistance is defined as “the ability of the host to prevent invasion (i.e., absence of a target receptor) or to clear the pathogen at the early stage of infection by mounting a protective immune response” (17). Disease tolerance is defined as “the mechanisms that decrease host susceptibility to tissue damage, or other fitness costs caused by pathogens or by the immune response” (18). Unlike resistance, disease tolerance does not necessarily imply direct effects on the pathogen load. Initially, it was observed that plants with a distinct genetic profile could tolerate an infection without affecting the pathogen load, which was termed “disease tolerance” (19, 20). More recently, the crucial role of disease tolerance in invertebrates and vertebrates against infectious diseases has been recognized (21, 22). The term disease tolerance is not to be confused with immunological tolerance, which is defined as unresponsiveness to self-antigens (18). Evidence of tolerance can potentially be observed in the host genome, with genetic variants that confer disease tolerance displaying positive or advantageous selection (23). However, the genetic *loci* influencing tolerance induction and the primary molecular and cellular mechanisms underlying disease tolerance in asymptomatic individuals are still largely unknown.

*Mycobacterium tuberculosis* and MAP have coevolved with humans and animals infrequently compromising host survival. Although this has been attributed to host resistance, our understanding of the mechanisms associated with disease tolerance in the infected individuals who become asymptomatic and disease-free is very limited. Since disease tolerance does not affect the pathogen burden as resistance is expected to do, it does not provoke the pathogen to develop new strategies to evade the host immune response. In addition, pathogen prevalence induces a continuous selection in favor of beneficial genetic variants for disease tolerance. While improving host resistance could lead to disease eradication, this is unlikely if hosts are tolerant as they can harbor the pathogen without showing clinical symptoms and lesions (24). For endemic diseases that result in a high number of asymptomatic animals, it might be more beneficial to improve tolerance than resistance. Furthermore, whereas resistance mechanisms are often pathogen-specific (e.g. mobilization of specific immune cells), tolerance mechanisms that prevent or repair damage may offer protection for a wide range of pathogens. Ultimately, tolerant mechanisms determine the overall health of the host and its longevity. Mycobacteria have developed several strategies to evade the resistance mechanisms of the host and persist for long periods. Clinical signs associated with Mycobacterial infections emerge as the host homeostasis becomes compromised due to tissue damage. In this context, the induction and modulation of tolerance pathways are critical to control inflammation and minimize pathology.

The response to MAP infection is complex and heritable leading to differences between individuals which can be categorized as (i) susceptible host, individuals who progress to severe forms of the disease, (ii) resistant host, individuals able to eliminate the bacteria by inducing protective innate immune responses at the early stage of infection, (iii) tolerant host, if innate immunity is unable to eliminate MAP, the host initiates tolerance mechanisms to contain MAP infection and prevent tissue damage. In the current study, we searched for genetic loci associated with tolerance to PTB by using WGS data from infected Spanish Holstein cows with MAP detected by PCR and bacteriological culture but without lesions in gut tissues and regional lymph nodes (N=24). A PTB tolerant animal was PCR and culture-positive (infected) but displayed no lesions in gut tissues (no disease). Using this case population, three case-control approaches were designed including as control population: 1) cows with negative PCR and culture results and with PTB-associated focal lesions (N= 253), 2) cows with PTB-associated focal lesions irrespective of their PCR and culture result (N= 288), and 3) cows with negative ELISA-PCR and culture results and without lesions in gut tissues (N= 373). Variance components, heritability (h^2^) estimates, SNPs, quantitative trait loci (QTL), and candidate genes were calculated for the three case-control approaches. Subsequently, functional pathways were identified for the case-control study with the highest h^2^ estimate, the case-control 1 (h^2^ = 0.55). Estimated breeding values (EBVs) for this population were calculated and cross-validated. Since there is evidence that some allelic variants may contribute to tolerance to multiple diseases, the identified QTLs and candidate genes were compared with QTLs and candidate genes for bovine diseases, and with human candidate genes for CD, IBD, colorectal cancer, multiple sclerosis, type I diabetes mellitus, and rheumatoid arthritis.

## Materials and Methods

### Ethics Statement

Animals used in this study were not submitted to any *in vivo* experimentation and, therefore, no specific ethics committee authorization was required. The cows were slaughtered in the Bilbao and Donostia municipal slaughterhouses (Basque Country, Spain) from March 2007 to May 2010 under the pertinent Basque (Basque Government Decree 454/1994), Spanish (Spanish Government Law 32/2007 and Royal decree 731/2007), and European (Council Regulation No 1099/2009) legislation on animal welfare.

### Animals

The study population consisted of 685 culled Spanish Holstein cattle from several herds located on seven regions: Basque Country (N= 286, 41.75%), Catalonia (N= 157, 22.92%), Navarre (N= 147, 21.46%), Cantabria (N= 49, 7.15%), Aragon (N= 22, 3.21%), Castile and Leon (N= 16, 2.34%), La Rioja (N= 5, 0.73%) and Asturias (N=3, 0.44%). All cows were 18 months years or older (5.6 years mean age).

### Histopathological Examination, Tissue PCR and Bacteriological Culture, and ELISA

Post-mortem tissue sampling, PCR, bacteriological culture, and ELISA were previously performed (25). For the histopathological analysis of gut tissues standardized protocols were used (5). Briefly, samples from the ileocecal lymph node, jejunal lymph node, ileocecal valve (ICV), jejunum, and terminal ileum were collected from each animal and fixed in 10% neutral buffered formalin for 72 h, dehydrated, embedded in paraffin, and cut into 4 μm sections. Several sections were mounted on microscope slides, stained with hematoxylin and eosin (HE) and with Ziehl-Neelsen (ZN), and examined. PTB-associated lesions were classified in focal, multifocal, and diffuse lesions as previously described (5). More specifically, PTB-associated focal lesions were defined according to their morphology and localization. They appear mainly in the lymph nodes (ileal and jejunal), characterize by the presence of small granulomas formed by 5 to 30 macrophages with undetected or low MAP load. Since similar focal lesions have been reported in experimental MAP infections and in the early stages of natural MAP infections in cattle (26–28), they were considered to represent initial forms of PTB. A pool (2 gr) of ICV, distal ileum and the jejunal caudal lymph nodes was decontaminated with 38 ml of hexadecyl pyridinium chloride at a final concentration of 0.75% (Sigma, St. Louis, MO) and homogenized in a stomacher blender. After 30 min of incubation at room temperature, 15 ml of the suspension was transferred to a new tube and incubated overnight for decontamination and sedimentation. Approximately, 200 µl of the suspension was taken and inoculated in duplicate Herrold´s egg yolk (Becton Dickinson, Franklin Lakes, NJ, USA) and Lowenstein-Jensen media (Difco, Detroit, MI, USA), both supplemented with 2 mg/L of mycobactin J (Allied Monitor, Fayette, MO, USA). If one or more MAP colonies were observed in any of the four medium slants the tissue culture was considered positive. Bacterial load in tissues was classified as low (<10 CFU/g), medium (between 10 to 50 CFU/g), or heavy (>50 CFU/gr). A second aliquot from the same tissue homogenates was used for DNA isolation and MAP IS900 amplification (Adiagene, Saint Brieuc, France). PCR amplifications were performed on an ABI Prism 7000 Sequence Detection System (Applied Biosystems, Foster City, CA, US). Samples with a threshold cycle (Ct) below 40.0 were considered positive. Antibody production against MAP in serum samples was tested using a two-step ELISA PTB antibody screening and verification kit (IDEXX Laboratories, Inc., Westbrook, ME, USA) according to the manufacturer´s instructions. The results were categorized as positive according to the sample-to-positive control ratio defined by the manufacturer.

### Genotyping and Imputation

DNA was extracted from peripheral blood samples collected from the study population at the time of slaughter and genotyped with the EuroG MD Bead Chip at the molecular genetic laboratory service of the Spanish Federation of Holstein Cattle (CONAFE). The InfiniumTM iScan software (Illumina, San Diego, CA) was used for allele assignation. The individual genotypes were previously phased and imputed to WGS level as previously described (15). Briefly, individual genotypes were phased using Eagle 2.4 (29) and imputed with minimac4 (30) to the Bovine HD Bead Chip using a reference panel of 1,278 *Bos taurus* from Run7.0 of the 1,000 Bull Genomes project and 581,712 SNPs (ASR-UCD1.2). Imputation to WGS level was then undertaken using a reference population of 2,333 *Bos taurus* from Run6.0 of the 1,000 Bull Genomes project (31). All the SNPs passed a call rate > 0.80. After filtering out SNPs with minimum allele frequency (MAF) < 0.01, we obtained 13,881,067 SNPs.

### Case-Control Studies, Variance Components, Standard Errors (SE), and h^2^ Estimates

A PTB tolerant animal was defined as an animal naturally infected with MAP that could harbor the pathogen without showing lesions in gut tissues that could be observed after the histopathological analysis of gut tissues. More specifically, animals with positive PCR and culture results but without PTB-associated lesions in gut tissues (N= 24). Using this case population, three case-control approaches were designed including as control population: 1) cows with negative PCR and culture results and with PTB-associated focal lesions (N= 253), 2) cows with PTB-associated focal lesions irrespective of their PCR and culture result (N= 288), and 3) cows with negative ELISA-PCR and culture results and without lesions in gut tissues (N= 373). The variance components, standard errors (SE), and h^2^ estimates explained by all the SNPs of each case-control population were calculated using the genome-wide complex trait analysis (*GCTA*) software 1.93.2, according to the following formula:


$$h^{2}=\frac{\sigma_{G}^{2}}{\sigma_{G}^{2}+\sigma_{e}^{2}}$$

where $\sigma_{G}^{2}$ is the variance explained by all the SNPs and $\sigma_{e}^{2}$ is the residual variance (32).

### Genome-Wide Association Study (GWAS)

In the current study, imputed genotypes (15) and the results of the diagnostic tests (25) were analyzed using the mixed linear model association analysis of the *GCTA* 1.93.2 software (32). Briefly, the model is *y = a + bx + g + e*, where *y* is the phenotype, *a* is the mean term, *b* is the additive effect (fixed effect) of the candidate SNP to be tested for association, *x* is the SNP genotype indicator variable coded as 0, 1 or 2, *g* is the polygenic effect (random effect) assumed to be distributed as N~(0, $\sigma_{G}^{2}$ ), and *e* is the residual assumed to be distributed as N~(0, $\sigma_{e}^{2}$ ). Age was included as a covariate in the analysis. After the GWAS, the SNPs with R^2^ values higher than 70% were retained. To account for multiple testing, a 5% genome-wide false discovery rate (FDR) was used. As strong evidence for association, a level of significance (*P* < 5 × 10^-7^) appropriate for genome-wide analysis was established according to the Welcome Trust Case Control Consortium (33). The inflation factor (*λ*) and quantile-quantile plots were calculated to compare the observed distributions of –log (*P*-values) to the expected distribution under the no association model. The regression coefficients (*b*) calculated using the *GCTA 1.93.2* software represents “the estimated increase in the log odds of the outcome per unit increase in the value of the exposure. In other words, the exponential function of *b* is the odds ratio (OR) associated with a one-unit increase in exposure” (34). In addition, the OR and their 95% confidence intervals (CI) for the SNPs associated with PTB tolerance (*P* < 5 × 10^-7^) were calculated using logistic regression analysis with the WGassociation function of *SNPassoc 1.9.2* (35).

### SNPs, QTLs, and Candidate Genes Identification

Localization of SNPs and QTLs and identification of candidate genes was performed using the ARS-UCD1.2 reference genome as previously described (15, 16). The genomic localization of the identified SNPs was determined using the Ensembl Variant Effect Predictor (VEP). QTLs were defined based on SNPs on linkage disequilibrium (LD) patterns with SNPs that surpassed the threshold (*P* < 5 × 10^-7^) in a given chromosome. Pairwise LD (r^2^ values) among all SNPs within 500,000 base pairs upstream and downstream of the suggestive SNP were calculated using PLINK 1.9. The beginning and end of each QTL were defined by the SNPs that were furthest upstream and downstream of the suggestive SNP and that had an r^2^ ≥ 0.9 (36, 37). Overlapping QTL regions were merged and considered as a single QTL. The defined QTL regions we further investigated for the presence of candidate genes within 50,000 base pairs to each side of the defined QTL using Ensembl (https://www.ensembl.org). The identified QTLs and candidate genes were compared with reported QTLs for bovine diseases (http://www.animalgenome.org) and with human candidate genes previously identified for CD, IBD, colorectal cancer, multiple sclerosis, type I diabetes mellitus, and rheumatoid arthritis (https://www.ebi.ac.uk/gwas). To further investigate the function of the identified candidate genes, we uploaded the 98 genes into the Innate DB database for innate immunity genes (https://innatedb.com).

### Gene Ontology (GO) and Pathway Enrichment Analysis

Candidate genes were investigated for significant enrichment of GO categories and Kyoto Encyclopedia of Genes and Genomes (*KEGG*) pathways using the *Cluster Profiler Bioconductor* package 3.10.1 (38). To account for multiple testing, *P*-adjust values were estimated.

### Estimated Breeding Values (EBVs) and Cross-Validation of the Genomic Predictions

The *GBLUP* (genomic best linear unbiased prediction) model (39) of *GCTA 1. 93. 2.* was used to estimate the breeding value of each individual of the study population attributed to the aggregative effect of the SNPs with evidence of association with the phenotype; *P* < 5 × 10^-5^ and *P* < 5 × 10^-7^, respectively. The accuracy of the genomic predictions was cross-validated in a validation population containing animals with a tissue culture positive result and without lesions in gut tissues (cases, N= 10) and animals with PTB-associated lesions in gut tissues (controls, N= 190). Cross-validation was performed using a 5-fold strategy. Briefly, phenotypes were masked (i.e., set to missing) for all the animals in a random 20% of the validation population and both phenotypes and genotypes for all other animals (i.e. training data set) were used to predict EBVs of individuals from the omitted group (i.e. testing dataset). This cross-validation process was iterated five times, such that each animal´s phenotype was masked at least once to generate its EBV. The ability of the group to determine whether or not each masked animal has a PTB tolerant phenotype was measured by the area under the receiver operating curve (AUC) for each model in each simulation using the ROCR package in R (40). The ROC curve is a graphical plot of the sensitivity, or true vs. false positive rate (1-specificity). The AUC would be 0.5 if its discrimination changes based on random chance, whereas if the model is perfectly able to determine the phenotype of each animal, the area under the curve would be 1.0 (41).

## Results

### Data, Variance Components, and h^2^ Estimates

The Spanish Holstein cows included in our study were previously tested by tissue PCR and bacteriological culture, ELISA, and histopathological analysis of gut tissues (25). Genotypes from the study population were previously obtained and imputed to WGS (15). In the current study, the associations between genotypes and PTB tolerance were investigated by using the genotypes from infected cows with a positive PCR and culture result and without lesions in gut tissues and regional lymph nodes (N= 24). All these animals had a low bacterial load in gut tissues (< 10 CFU/g) and a negative ELISA result. Using this case population, three case-control approaches were designed using as control population 1) cows with negative PCR and culture results and with PTB-associated focal lesions (N= 253), 2) cows with PTB-associated focal lesions irrespective of their PCR and culture result (N= 288), and 3) cows with negative ELISA-PCR and culture results and without lesions in gut tissues (N= 373). **Table 1** summarizes the number of cases and controls, variance components, and h^2^ estimates, for each of the three case-control approaches. The h^2^ estimates were 0.55, 0.44 and 0.18 for case-control studies 1, 2, and 3, respectively (**Table 1**). The small differences in h^2^ estimates (0.55 and 0.44) between the case-control studies 1 and 2 suggest that there would be little bias due to the use of either sample as the control group. The highest h^2^ estimate (h^2^ = 0.55) was obtained when cows with negative PCR and culture results and with PTB-associated focal lesions (N= 253) were the control population, highlighting the importance of the selection of control individuals from the lower extremity of the relevant trait distribution to increase sensitivity. The b-values ranged from 0.57 to 0.94 for the first case-control study and the range was 0.27-0.59 for the second case-control study providing more evidence that the most powerful approach was the first case-control study.

**Table 1:** Number (N) of cases and controls, variance components, standard errors (SE), and h^2^ estimates.

Study Code	Phenotype	Lesion, Tests results	N	SNPs FDR ≤ 0.05 P < 5 × 10^-7^	Additive genetic variance ( $\sigma_{G}^{2}$ )	Residual variance ( $\sigma_{e}^{2}$ )	Heritability (h^2^)
1	Case	No lesion, PCR/Culture Positive	24	40	0.044427	0.036225	0.55
	Control	Focal lesion, PCR/Culture Negative	253				
							
2	Case	No lesion, PCR/Culture Positive	24	136	0.032145	0.039855	0.44
	Control	Focal lesion	288				
							
3	Case	No lesion, PCR/Culture Positive	24	126	0.010334	0.046331	0.18
	Control	No lesion, ELISA/PCR/Culture Negative	373				

According to the Welcome Trust Case Control Consortium, the threshold was P < 5 x 10^-7^. FDR, false discovery rate.

### GWAS

GWAS using the imputed WGS datasets (15) and the phenotypes defined in the three case-control approaches were performed. The number of SNPs that surpassed the *P* < 5 x 10^-7^ threshold is presented in **Table 1**. Three SNPs on BTA25 (11.89-12.93 Mb) overlapped with the three case-control approaches suggesting an important link of this BTA25 region with the PTB tolerant animals. Twenty-eight SNPs were associated with the case-control studies 1 and 2, and four with the case-control studies 1 and 3.

**Figure 1:**
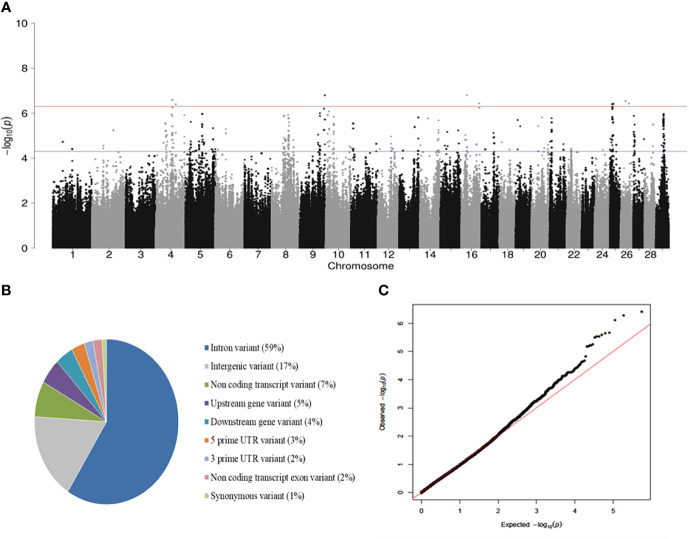
GWAS for PTB tolerance. **(A)** Manhattan plots showing –log_10_ (*P*-values) of association between every single SNP and phenotype. The associations between host genetic and tolerance were investigated using the genotypes from 277 Spanish Holstein cows with two distinct phenotypes: cases) positive PCR and culture result without lesions (N = 24), and controls) negative PCR and culture result with PTB-associated lesions (N = 253). Each dot represents the result from the test association for a single SNP. The horizontal red line shows –log_10_ (5 × 10^-7^) and the blue line –log_10_ (5 × 10^-5^). **(B)** Genomic distribution of the SNPs associated (FDR≤ 0,05, *P* < 5 × 10^-7^) with tolerance according to the Ensembl Variant Effect Predictor (VEP). **(C)** Quantile-quantile plot compared the observed distribution of –log (*P*-values) to the expected values under the null hypothesis.

A total of 40 (*P* < 5 x 10^-7^) SNPs were associated with PTB tolerance defined using the case-control approach with the highest hereditability (h^2^ = 0.55). In this approach, animals were considered cases when they were PCR and culture positives but they did not show lesions in gut tissues (N= 24). Controls were PCR and culture negatives with focal PTB-associated lesions in gut tissues (N= 253). A Manhattan plot showing –log_10_ (*P*-values) of association between every single SNP and PTB tolerance is presented in **Figure 1A**. The 40 SNPs associated with tolerance (FDR ≤ 0.05, *P* < 5 × 10^-7^) were located on the *Bos taurus* chromosomes BTA4, BTA9, BTA16, BTA25, and BTA26. Most of these SNPs (59%) were located in intronic regions (**Figure 1B**). A quantile-quantile plot was generated (**Figure 1C**). The plot showed a distribution close to the expected line (λ_median_= 1.03), indicating that significant values were not overestimated due to population stratification or cryptic relatedness. The regression coefficients (*b*-values) for the 40 SNPs associated with PTB tolerance (*P* < 5 × 10^-7^) were all positive (data not shown). In addition, the OR and 95% confidence intervals (CI) calculated for the 40 SNPs were > 1 indicating that the animals with minor alleles had a higher probability of being tolerant (**Table 2**). The two SNPs with the highest OR (25.89 and 20.71) were located on BTA4 and BTA9. Interestingly, three of the 12 SNPs with OR= 15.86 and located on BTA25 (11.89-12.93 Mb) were common to the three case-control approaches.

**Table 2:** Frequency of cases and controls and odds ratio (OR) under the additive genetic model for the 15-top SNPs associated with tolerance (FDR ≤ 0.05, *P* < 5 × 10^-7^).

Chromosome	SNP position	SNP ID	Genotype	Cases (%)	Controls (%)	OR (95% CI)
4	805,731		C/C	20 (83.3)	251 (99.2)	1.0
			T/C	4 (16.7)	2 (0.8)	25.89 (4.4-152.5)
9	101,497,090	rs436366135	A/A	19 (79.2)	250 (98.8)	1.0
			G/A	5 (20.8)	3 (1.2)	20.71 (4.38-97.94)
25	12,394,597	rs109954622	G/G	18 (75.0)	248 (98.0)	1.0
			A/G	6 (25.0)	5 (2.0)	15.86 (4.23-59.54)
25	12,396,223	rs381977996	C/C	18 (75.0)	248 (98.0)	1.0
			T/C	6 (25.0)	5 (2.0)	15.86 (4.23-59.54)
25	12,411,628	rs379340537	C/C	18 (75.0)	248 (98.0)	1.0
			T/C	6 (25.0)	5 (2.0)	15.86 (4.23-59.54)
25	12,413,134	rs379127550	C/C	18 (75.0)	248 (98.0)	1.0
			A/C	6 (25.0)	5 (2.0)	15.86 (4.23-59.54)
25	12,415,339	rs109985656	G/G	18 (75.0)	248 (98.0)	1.0
			A/G	6 (25.0)	5 (2.0)	15.86 (4.23-59.54)
25	12,415,928	rs382534620	G/G	18 (75.0)	248 (98.0)	1.0
			C/G	6 (25.0)	5 (2.0)	15.86 (4.23-59.54)
25	12,427,653	rs42060371	T/T	18 (75.0)	248 (98.0)	1.0
			C/T	6 (25.0)	5 (2.0)	15.86 (4.23-59.54)
25	12,428,267	rs42060369	T/T	18 (75.0)	248 (98.0)	1.0
			C/T	6 (25.0)	5 (2.0)	15.86 (4.23-59.54)
25	12,428,660	rs42060368	T/T	18 (75.0)	248 (98.0)	1.0
			C/T	6 (25.0)	5 (2.0)	15.86 (4.23-59.54)
25	12,429,377	rs42060367	T/T	18 (75.0)	248 (98.0)	1.0
			C/T	6 (25.0)	5 (2.0)	15.86 (4.23-59.54)
25	12,429,409	rs42060366	A/A	18 (75.0)	248 (98.0)	1.0
			C/A	6 (25.0)	5 (2.0)	15.86 (4.23-59.54)
25	12,430,197	rs109193429	T/T	18 (75.0)	248 (98.0)	1.0
			C/T	6 (25.0)	5 (2.0)	15.86 (4.23-59.54)
16	72,010,469	rs210839825	G/G	18 (75.0)	248 (98.0)	1.0
			G/C	6 (25.0)	5 (2.0)	15.72 (4.25-58.24)

### SNP Variants, QTLs, and Candidate Genes Identification

The SNPs surpassing the significance threshold (*P* < 5 × 10^-7^), *P*-values, and candidate genes located within the defined QTLs are presented in **Table 3** for the case-control 1, and in **Supplementary Tables 1** **,** **2** for the case-control studies 2 and 3. Five of the nine QTLs identified in the case-control study 1 and located on BTA4, BTA9, BTA25 (8.90-9.99 Mb, 11.89-12.93 Mb), and BTA26 were associated with the case-control study 2 as well. Case-control studies 1 and 3 shared two QTLs on BTA16 (71.51-72.51 Mb) and BTA25 (11.89-12.93 Mb). According to the Welcome Trust Consortium “a study design using hypercontrols (that is, selection of control individuals from the lower extremity of the relevant trait distribution) would generally be the most powerful approach in a study focusing on one disease” (33). Therefore, we focused on the results of the case-control study 1 with the highest h^2^ estimate (0.55) and *b*-values (0.57-0.90). No differences in age between the cases (74.73 ± 38.13 months) and controls (68.29 ± 25.79 months) (P= 0.2659) included in the case-control study 1 were observed.

**Table 3:** QTLs surpassing the significance threshold (*P* < 5 × 10^-7^) for evidence of an association with tolerance.

BTA^1^	QTL start (bp)	QTL end (bp)	P-value most significant SNP	SNP position ^2^	SNP ID	Annotation	Genes in QTL ^3^	N° ofsignificant SNPs in QTL
16	21307714	22307714	1,58E-07	21807714			*SPATA17, RRP15, TGFB2, U6*	1
9	100997090	101997090	1,60E-07	101497090	rs436366135	Intron	*TBXT,U6, SFT2D1, MPC1, RPS6KA2, RNASET2, CEP43, CCR6, GPR31, U4, TTLL2, UNC93A, ENSBTAG0000051317, ENSBTAG00000050267, ENSBTAG00000054087, ENSBTAG00000053924*	1
4	66265303	67309158	2,55E-07	66765303	rs382143408	Intron	*PLEKHA8, SCRN1, FKBP14, WIPF3, PRR15, CHN2, CPVL, TRIL, CREB5, ENSBTAG00000054573*	8
26	20050339	21059555	2,92E-07	20559555	rs381461650	Intergenic	*CNNM1, GOT1, U6, NKX2-3, SLC25A28, ENTPD7, COX15, CUTC, ABCC2, DNMBP, CPN1, ERLIN1, CHUK, ENSBTAG00000052639, ENSBTAG00000050951, ENSBTAG00000045082, ENSBTAG00000044887, ENSBTAG00000033021, ENSBTAG00000023939*	2
16	71510469	72510469	3,61E-07	72010469	rs210839825	Intron	*DTL, INTS7, LPGAT1, NEK2, SLC30A1, RD3, TRAF5, RCOR3, KCNH1, ENSBTAG00000048992, ENSBTAG00000048697, ENSBTAG00000050969, ENSBTAG00000053608*	1
26	33908564	34908564	3,69E-07	34408564	rs210720477	Intergenic	*HABP2, NRAP, CASP7, PLEKHS1, NHLRC2, DCLRE1A, ADRB1, CCDC186, TDRD1, VWA2, AFAP1L2, bta-mir2285dg, ABLIM1, ENSBTAG00000051735, ENSBTAG00000053724*	1
25	11894597	12930197	3,81E-07	12394597	rs109954622	Intergenic	*ERCC4*	12
25	8908535	9991143	3,93E-07	9408535	rs380720091	Intron	*U6, ATF7IP6, EMP2, TEKT5, NUBP1, TVP23A, CIITA, DEXI, CLEC16A, SOCS1, RMI2, TNP2, PRM1, PRM2, PRM3, ENSBTAG00000052111*	13
4	80073101	81073101	4,02E-07	80573101	rs379792050	Intron	*SUGCT, MPLKIP, CDK13, ENSBTAG00000049322*	1

^1^QTL location, ^2^SNP location in the genome, ^3^Candidate genes located within the identified QTL.

The 40 SNPs associated with PTB tolerance resided within 9 QTLs on 5 chromosomes including BTA4, BTA9, BTA16, BTA25, and BTA26 (**Table 3**). Twenty-five SNPs (62.5%) were located on BTA25 (8.90-12.93 Mb) highlighting the important association of this region with the phenotype. The BTA25 and BTA26 harbored 4 of the 9 identified QTLs. The QTL that harbored the most genome-wide significantly-associated SNP (*P*= 1.58E-07) was located on BTA16 (21.30-22.30 Mb). By examining the available cattle QTL database (**Table 4**), we observed that this QTL together with a QTL located in BTA26 (33.90-34.90 Mb) overlap with two QTLs previously reported in the literature that were associated with PTB susceptibility; QTL:14860 and QTL:14880, respectively (42). The largest QTL was located on BTA25 (8.90-9.99 Mb) and contained 13 SNPs and 16 candidate genes. This QTL overlap with QTLs associated with clinical mastitis (QTL:1751) (43) and somatic cell score (QTL:4993, QTL:52494) (44, 45). A second QTL identified on BTA25 (11.89-12.93 Mb) overlap with QTLs associated with bovine tuberculosis susceptibility (QTL:96564) (46), clinical mastitis (QTL:1751) (43), and somatic cell score (QTL:177968, QTL:32484, QTL:32485, QTL4993, QTL:52521) (45, 47, 48). The two QTLs identified in the current study on BTA16 and one of the QTLs identified on BTA26 (20.05-21.05 Mb) overlap with QTLs associated with length of productive life highlighting the association of this trait with PTB and health (45). Interestingly, a QTL identified in our study on BTA26 (33.90-34.90 Mb) overlaps with a QTL associated with bovine diarrhea virus persistent infection (QTL:66096) (49).

**Table 4:** Overlapping of the identified QTLs with regions associated with other cattle diseases and relevant traits.

Identified QTLs	PTB susceptibility	Bovine tuberculosis susceptibility	Clinical Mastitis	Somatic cell score	Tick resistance	Length of productive life	Bovine diarrhea virus infection
4:66265303-67309158					QTL:135872		
4:80073101-81073101		QTL:96207					
9:100997090-101997090							
16:21307714-22307714	QTL:14860			QTL:48185, QTL:48208,		QTL:48183, QTL:48205	
16:71510469-72510469				QTL:48470		QTL:48467	
25:8908535-9991143			QTL:1751	QTL:4993, QTL:52494			
25:11894597-12930197		QTL:96564	QTL:1751	QTL:177968, QTL:32484, QTL:32485, QTL:4993, QTL:52521			
26:20050339-21059555			QTL:161603			QTL:52778	
26:33908564-34908564	QTL:14880			QTL:32489			QTL:66096

The candidate genes associated with MAP tolerance that were identified in the current study had not been associated with bovine PTB, tuberculosis, or mastitis before. **Table 3** presents a list of the 98 candidate genes. We identified candidate genes in all the QTLs. Only one candidate gene, the *Excision Repair 4 (ERCC4)*, was identified on one of the QTLs located on BTA25 (11.89-12.93 Mb). *ERCC4* is a DNA repair endonuclease (*XPF)*. Together with *ERCC1*, *XPF* forms the *ERCC1-XPF* enzyme complex that participates in the nucleotide excision DNA repair (NER) pathway, a highly conserved mechanism that recognizes and removes helical distortions through the genome.

### Functional GO and Metabolic Pathways

We identified 7 GOs and 2 metabolic pathways significantly enriched in the PTB tolerant animals. As seen in **Table 5**, two biological processes (BPs) related to DNA packaging and DNA conformation change were significantly enriched (*P* ≤ 0.05). We observed that five cellular compartments (CC) associated with DNA packaging such as nucleosome, chromatin, chromosome, DNA packaging complex, and protein-DNA complex were also enriched. Gene members of the *Transition protein 2* (*TNP2*) and *Protamine* (*PRM*) family such as *PRM1, PRM2*, and *PRM3* were common to these seven GOs. *TNP2* plays a key role in the replacement of histones to *PRMs* during spermatogenesis and *PRMs* compact DNA into a highly condensed, stable, and inactive complex. Pathway analysis on the 98 candidate genes revealed a significant enrichment of the toxoplasmosis (bta05145; *TGFβ2/CHUK/CIITA/SOCS1*) and *TNF*-signaling (bta04668; *TRAF5/CREB5/CASP7/CHUK*) pathways with four proteins matching each route. Interestingly, the *nuclear Factor NF-κβ Inhibitor Kinase Alpha* (*CHUK or IkBKA*), a key molecule in the *NF- κβ* signaling pathway, was enriched in both pathways (*P* ≤ 0.01).

**Table 5:** Results of the gene ontology and pathway analysis using the identified candidate genes.

Category	ID	Description	*P* adjust	Genes	Genes ratio
BP	GO:0006323	DNA packaging	0.0022	*TNP2/PRM1/PRM2/PRM3*	4/20
	GO:0071103	DNA conformation change	0.0220	*TNP2/PRM1/PRM2/PRM3*	4/20
CC	GO:0000786	Nucleosome	0.0001	*TNP2/PRM1/PRM2/PRM3*	4/20
	GO:0044815	DNA packaging complex	0.0001	*TNP2/PRM1/PRM2/PRM3*	4/20
	GO:0032993	Protein-DNA complex	0.0010	*TNP2/PRM1/PRM2/PRM3*	4/20
	GO:0000785	Chromatin	0.0079	*TNP2/PRM1/PRM2/PRM3*	4/20
	GO:0005694	Chromosome	0.0439	*INTS7/TNP2/PRM1/PRM2/PRM3*	5/20
Pathway	bta05145	Toxoplasmosis	0.0126	*TGFB2/CHUK/CIITA/SOCS1*	4/21
	bta04668	TNF signaling pathway	0.0126	*TRAF5/CREB5/CASP7/CHUK*	4/21

### Estimated Breeding Values (EBVs) and Cross-Validation of the Genomic Predictions

**Figure 2:**
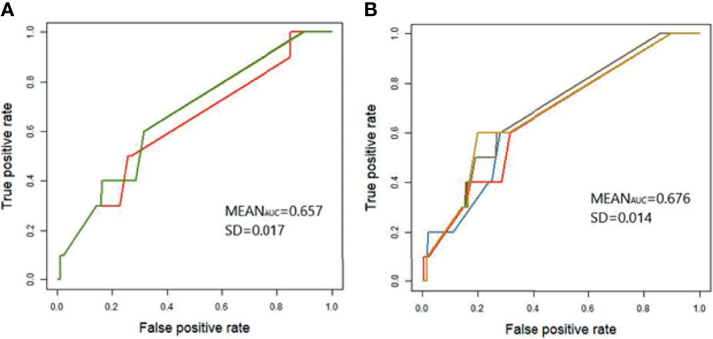
Receiver Operating Characteristics (ROC) curve for application of genomic predictions from the study population to the validation population. *GBLUP* models were developed using the 142 **(A)** and 40 **(B)** SNPs with evidence of association with the case-control population 1; *P* < 5 × 10^-5^ and *P* < 5 × 10^-7^, respectively. Genetic predictions were cross-validated in the validation population using a five-fold strategy as described in *Materials and Methods*. Each curve represents one of the five generated models and models might occasionally overlap. The average AUC and standard deviation (SD) across the five cross-validations are included in each figure.

The *GBLUP* model (39) was used to estimate breeding values of each individual in the study population attributed to the aggregative effect of the 142 and 40 SNPs with evidence of association with the phenotype; *P* < 5 × 10^-5^ and *P* < 5 × 10^-7^, respectively. The genomic predictions were cross-validated using a 5-fold strategy as described in *Materials and Methods*. The efficacy of the models was examined by ROC analysis, which provides a measure of the probability of the model´s successful classification of samples (**Figure 2**). The AUC (± SD) of the models generated using the 142 (AUC= 0.657 ± 0.017) and 40 (AUC= 0.676 ± 0.014) SNPs were very similar which suggested that the model developed with the 40 most significant SNPs is a good classifier. The cut-off point for the EBVs in the study population was obtained with the Statistical Analysis in Epidemiology (Epi) R package (cut-off= 0.0356).

## Discussion

Understanding tolerance pathways is a significant challenge due to the difficulty in determining the tolerance phenotype from field data. The identification of genetic variants underlying variation in tolerance to pathogens requires both great genome coverage and measuring disease outcomes that sometimes are not readily measured. In this study, we aim to elucidate cellular and molecular mechanisms responsible for disease tolerance using cattle naturally infected with MAP that can harbor the pathogen without showing lesions in gut tissues. Although a previous GWAS identified loci associated with tolerance to Johne´s disease (50), our GWAS is the first to complete an analysis of the genetic markers associated with tolerance to PTB using WGS data and epidemiological data based on three diagnostic tests. Results from the current study do not corroborate those of Zanella et al. (50) who identified a tolerance-associated region of 6.5 Kb on BTA15 which we did not identify here. This might be due to differences in the phenotypes used. In the study by Zanella et al, tolerance measured the relationship between infection intensity (level of MAP in the tissues) and fitness (level of MAP fecal shedding), but to define tolerance is critical to measure disease outcomes, not just infection burden. In our study, PTB tolerant cows are infected animals with positive PCR and bacteriological culture results but without lesions in gut tissues. Histopathological analysis usually detects more infected animals than bacteriological culture and PCR (5). However, in the study by Gonzalez et al, 3/116 (2.5%) of the animals were PCR and culture positives but without lesions in gut tissues. Similarly, in our study population (N=983) (15), 24 animals without lesions in gut tissues were PCR and culture positives (2.4%). The highest h^2^ estimate (h^2^ = 0.55) was obtained when cows with negative PCR and culture results and with focal lesions (N= 253) were the control population, highlighting the importance of the selection of control individuals from the lower extremity of the relevant trait distribution to increase sensitivity. In a large number of PTB-associated focal lesions, MAP could not be detected due to the low numbers of bacteria that cannot be easily detected by bacteriological culture or PCR (25–28, 51, 52). In humans, inflammatory bowel disease is solely defined by pathological findings since no causal pathogen has been unanimously recognized. Despite the small sample size of cases (N=24), the Manhattan plot showed loci with associations with the underlying SNPs. A total of 40 novel SNPs (FDR ≤ 0.05 and *P* < 5 × 10^-7^) defining 9 QTLs and 98 candidate genes were associated with the tolerant cattle. All these SNPs had positive b-values and OR > 1 and were, therefore, associated with PTB tolerance. EBVs were estimated for the study population using the SNPs with a significant association with the phenotype (FDR ≤ 0.05) and the genetic predictions were subsequently cross-validated in a different population.

The 98 candidate genes identified in our study were compared with candidate genes previously reported in CD, IBD, colorectal cancer, multiple sclerosis, type I diabetes mellitus, and rheumatoid arthritis. Seven genes including *Glutamic-Oxaloacetic Transaminase 1 (GOT1), NK2 Homeobox 3* transcription factor (*NKX2-3*), *Solute Carrier Family 25 Member 8 (SLC25A28*), *Fibroblast Growth Factor Receptor 1 Oncogene Partner (CPE43*), *Chemokine Receptor-Like 3 (CCR6*), *C-Type Lectin Domain Containing 16A (CLEC16A)*, and *RecQ Mediated Genome Instability 2 (RMI2)*, were previously associated to rheumatoid arthritis. The last two genes, *CLEC16A and RMI2*, were also associated with CD, IBD, multiple sclerosis, and type I diabetes mellitus. *CLEC16A* is a regulator of mitophagy/autophagy and mitochondrial health and *RMI2* plays a critical role in homologous recombination-dependent DNA repair (53). *NKX2-3* and *SLC25A28* were associated with CD, IBD, colorectal cancer, type I diabetes mellitus, and rheumatoid arthritis. Five of the 98 candidate genes have relevant innate immunity functions including *CCR6*, the *nuclear Factor NF-κβ Inhibitor Kinase Alpha* (*CHUK or IkBKA*), *STAT-Induced STAT Inhibitor 1 or suppressor of cytokine Signaling 1* (*SOCS1*), *TNF Receptor Associated Factor 5* (*TRAF5*), *and caspase 7 (CASP7)*, a caspase involved in the activation cascade of caspases responsible for apoptosis.

**Figure 3:**
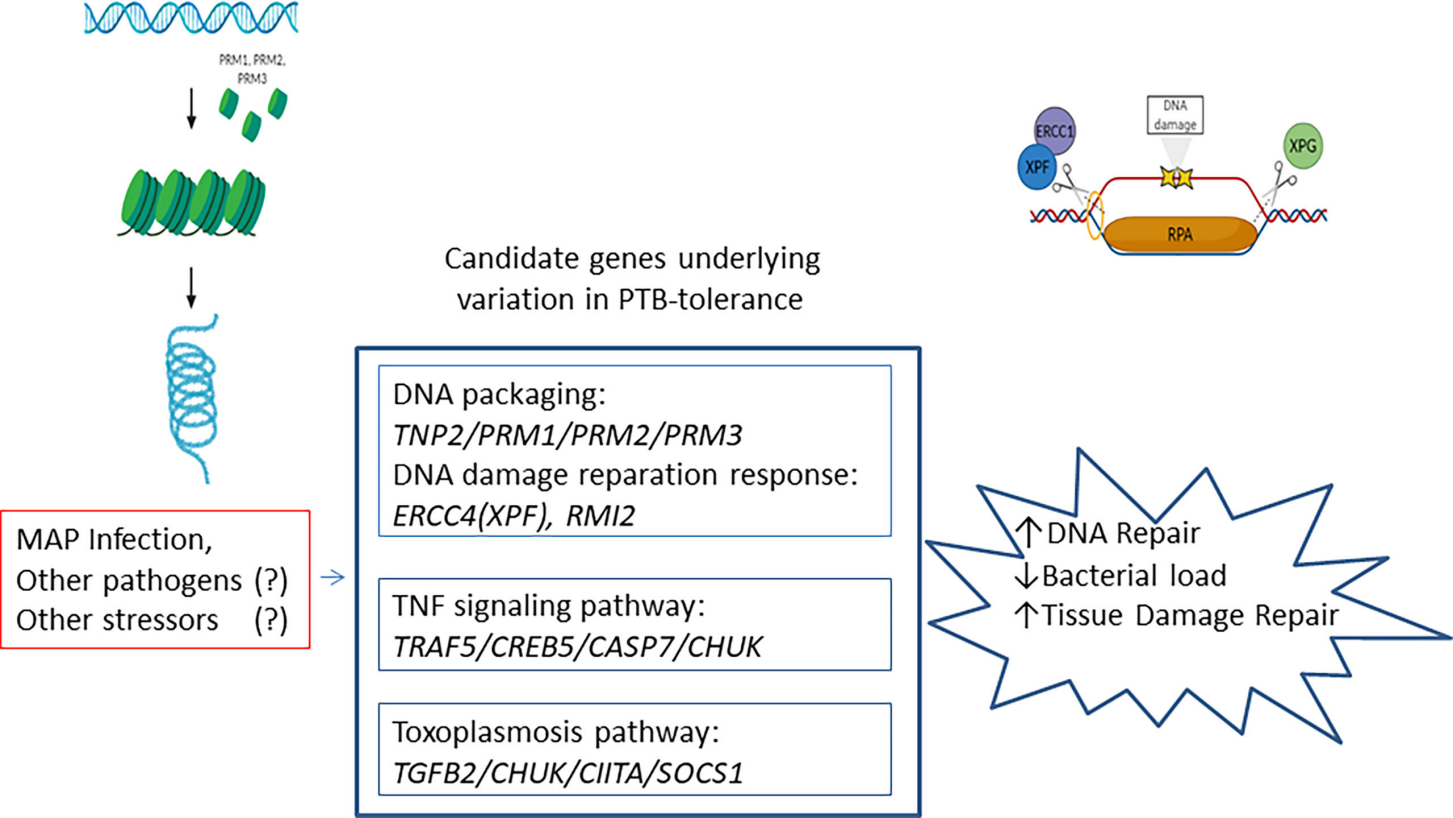
Schematic diagram illustrating the identified PTB-tolerance mechanisms. Candidate genes involved in DNA packaging, *TNF* signaling pathway, and toxoplasmosis were associated with the PTB tolerant individuals. These mechanisms play conserved and important roles in disease tolerance.

Although poorly understood, the mechanisms underlying disease tolerance appear to involve conserved stress and damage responses (54). When these responses fail, the default response becomes programmed cell death of infected cells which is coupled to their immediate phagocytosis by macrophages and subsequent polarization. This macrophage polarization is also associated with the production of cytokines such as *transforming growth factor β1* (*TGFβ1*) which can promote tissue regeneration. Consistent with these results, our pathways analysis with the 98 identified candidate genes revealed a significant enrichment of the DNA packaging (*TNP2/PRM1/PRM2/PRM3*), *TNF*-signaling pathway (*TRAF5/CREB5/CASP7/CHUK*), and toxoplasmosis (*TGFβ2/CHUK/CIITA/SOCS1)* in the tolerant animals (**Figure 3**). Upstream of the *TNP2, PRM1, PRM2*, and *PRM3* genes located on BTA25, we identified the *RMI2* gene which is critical for DNA damage repair and genome stability (53). Moreover, we identified 12 genetic variants on BTA25 (11.89-12.93 Mb) upstream of the *Excision Repair 4 (ERCC4)* gene. *ERCC4* forms the *ERCC1-XPF* enzyme complex that participates in the nucleotide excision DNA repair (NER) pathway. Altogether, these findings provide evidence for a link between PTB tolerance and genetic variants in candidate genes involved in DNA packaging, DNA damage repair response, innate immune responses, and pathogen persistence.

In most eukaryotic cells, nucleosomes are the basic structural units of chromatin formed by a histone octamer core. A unique chromatin remodeling process occurs extensively during spermatogenesis, which leads to more than 90% of core histones being replaced by transition proteins (*TNP*), and then *PRMs* (55, 56). The number of histones that are replaced by *PRMs* and the proportion of *PRM1/PRM2* are essential to keep chromatin structure. So if this proportion changes, the DNA is poorly packed and susceptible to damage (57). The specialized structure of the sperm chromatin-*PRM* has a dual function; first, to protect the DNA from damage during transport to the oocyte, and then to enable a rapid and complete unpacking of the undamaged paternal genome in the ooplasm (58). In the ooplasm, maternal factors rapidly access the paternal chromatin and begin the replacement of *PRM* with maternal histones (59). Any damage to the sperm DNA during the transition from the testicle to the oocyte cannot be repaired until the DNA is accessible for DNA repair systems in the ooplasm (58). On the other hand, oocytes are especially sensitive to DNA damage when they resume meiosis during the menstrual cycle. These DNA lesions, if not repaired correctly, can endanger the fitness and viability of the cell. Since DNA damage is one of the most important factors that can compromise the viability of the cell and/or organism and contribute to diseases, it is well recognized that individuals with enhanced DNA packaging and DNA damage responses have improved homeostasis and prolonged healthy lifespan (60).

Schneider et al., reported that mutants in the fly *TNF*–related molecule “Eiger” when challenged with a bacterial species, lived longer with similar pathogen titers (61). Similarly, we found a significant enrichment of candidate genes involved in the *TNF*-signaling pathway (*TRAF5/CREB5/CASP7/CHUK*) in the PTB tolerant animals. This pathway can trigger programmed cell death while also activating pro-survival responses *via* activation of *NF-κβ*. *TRAF5, CASP7*, and *CHUK or IkBKA*, have well-recognized roles in the innate immune responses. *CHUK* is a serine kinase that plays an essential role in the canonical *NF-κβ* signaling pathway. *CHUK* acts as part of the canonical *IKK* complex phosphorylating inhibitors of the *NF-κβ* on serine residues. In turn, free *NF-κβ* is translocated into the nucleus and activates the transcription of genes involved in immune response, growth control, or protection against apoptosis. *CASP7* is responsible for apoptosis, which is regarded as an important mechanism through which the host can restrict pathogen replication in infected cells and maximize disease tolerance*. TRAF5* is a member of the *TNF* receptor-associated factor (*TRAF*) family that mediates *TNF*-induced activation. *TNF-*signaling activates *NF-κβ* and *JNK* pathways.

Although the *TNF*-signaling pathway constitutes a critical host defense against Mycobacteria, its excess is also implicated in immunopathogenesis and activation of programmed necrosis pathway *via* induction of mitochondrial reactive oxygen species (ROS) that kills both bacteria and infected macrophages (62). Therefore, the innate immune response, while important to clear the pathogen, can also cause tissue damage. In addition to the *TNF* pathway, we detected enrichment of candidate genes involved in the toxoplasmosis (*TGFβ2/CHUK/CIITA/SOCS1)* pathway in the tolerant animals. Interestingly, *CHUK* was one of the candidate genes in the toxoplasmosis pathway which highlights the role of *CHUK* as an important modulator of the inflammatory response. As part of the non-canonical pathway of *NF-κβ* activation, *CHUK* participates also in the negative feedback of *NF-κβ* signaling pathway preventing *TNF*-mediated *RIPK1*-dependent cell death. While the canonical *NF-κβ* activation is associated with rapid and acute production of diverse pro-inflammatory mediators, the non-canonical *NF-κβ* signaling pathway is tightly regulated and associated with less acute, chronic inflammation, and with maintaining homeostasis. Consequently, the role of *CHUK* in controlling inflammation by activating or limiting the activation of *NF-κβ* may be timing and/or environmental dependent. Overall, the activation of the toxoplasmosis pathway in the tolerant animals might result in a lifelong and persistent MAP infection as seen in *Toxoplama gondii* infected hosts. *Toxoplasma gondii* and other intracellular parasites which typically establish themselves as long-term residents in mammalian hosts are often associated with elevated host *TGFβ* expression (63). Direct activation of the *signal transducer and activator of transcription 3* (*STAT3*) by the parasite enhances the anti-inflammatory function of *TGFβ* causing lifelong infection by establishing an anti-apoptotic environment. In addition, the role of *TGFβ2* in tissue healing and repair is well established promoting disease tolerance at later time points (64). We also found two novel candidate genes associated with PTB tolerance, the *Class II Major Histocompatibility Complex Transactivator* (*CIITA)* and the *suppressor of cytokine signaling* (*SOCS1).* Both genes are located on BTA25 upstream of the *RMI2/TNP2/PRIM1/PRM2/PRM3* region and adjacent to the *CLEC16A* gene, a regulator of phagosome-lysosome fusion during late mitophagy. *CIITA* is a positive regulator of class II major histocompatibility complex gene transcription and is the “master control factor” for the expression of these genes and T-cell activation. *SOCS1* is a member of the *STAT*-induced *STAT* inhibitor (SSI) family, with important roles in immune cell homeostasis and regulation of inflammation which is crucial to avoid an excessive inflammatory response and to minimize tissue damage.

## Conclusions

In this study, we explored the concept of disease tolerance in the context of a chronic intestinal infection. We were able to show that there is genetic variation associated with PTB tolerance. We revealed the genetic basis for PTB tolerance and the molecular pathways involved. Pathways analysis using the identified candidate genes revealed mechanisms underlying DNA packaging, innate immunity, and establishment of persistent infection that support tolerance to PTB. Strategies designed to improve diseases tolerance are expected to improve diseases control and to reduce the dependence on antimicrobials to kill pathogens, which in turn would reduce antimicrobial resistance. In livestock, the identification of genetic variants that regulate disease tolerance could help to breed for more tolerant cattle to a variety of diseases and likely to other environmental stressors as well.

## Data Availability Statement

The datasets presented in this study can be found in online repositories. The names of the repository/repositories and accession number(s) can be found in the article/**Supplementary Material**.

## Ethics Statement

Animals used in this study were not submitted to any *in vivo* experimentation and, therefore, no specific ethics committee authorization was required.

## Author Contributions

MC performed the GWAS and wrote the manuscript. PV, JG, and RJ participated in samples collection and phenotypic characterization of the animals. GB-B performed the functional analysis, estimation of breeding values and cross-validation, and helped with the figures. OG-R and AF supervised the imputation to WGS, the GWAS, and the cross-validation of the genomic predictions. MA-H conceived and coordinated the project, supervised the data analysis, and wrote the manuscript. All authors revised and approved the final manuscript.

## Funding

Financial support for this study was provided by a grant (RTI2018-094192-R-C21) funded by MCIN/AEI/10.13039/501100011033 and by FEDER, “Una manera de hacer Europa”. MC and GB-B have been awarded fellowships from INIA and MCIN/AEI/10.13039/501100011033 and “FSE Invierte en tu futuro”; grants FPI2016-00041 and PRE2019-090562, respectively.

## Conflict of Interest

The authors declare that the research was conducted in the absence of any commercial or financial relationships that could be construed as a potential conflict of interest.

## Publisher’s Note

All claims expressed in this article are solely those of the authors and do not necessarily represent those of their affiliated organizations, or those of the publisher, the editors and the reviewers. Any product that may be evaluated in this article, or claim that may be made by its manufacturer, is not guaranteed or endorsed by the publisher.
