# Supplementary material for: A Genome-Wide Association Study for Tolerance to Paratuberculosis Identifies Candidate Genes Involved in DNA Packaging, DNA Damage Repair, Innate Immunity, and Pathogen Persistence
Source: Front Immunol. 2022 Apr 6;13:820965. doi: 10.3389/fimmu.2022.820965 (PMC9019162; doi:10.3389/fimmu.2022.820965)
Supplement: Supplementary file 1 [file Table_1.docx]

**Supplementary Table 1.** QTLs surpassing the significance threshold (P < 5 × 10^-7^) for evidence of an association with the case-control study 2.

| **BTA^1^** | **QTL start (bp)** | **QTL end (bp)** | **P-value most significant SNP** | **SNP position^2^** |  | **Annotation** | **Genes in QTL^3^** | **Nº of significant SNPs in QTL** |
| --- | --- | --- | --- | --- | --- | --- | --- | --- |
| 4 | 80073101 | 81073101 | 4,93915E-08 | 80573101 | rs379792050 | Intron | SUGCT, MPLKIP, CDK13, ENSBTAG00000049322 | 1 |
| 4 | 114739448 | 1,16E+08 | 2,11338E-07 | 1,15E+08 | rs110176296 | Intergenic | KMT2C, CCT8L2, XRCC2, ACTR3B, 5S_rRNA, ENSBTAG00000019597, ENSBTAG00000009002 | 1 |
| 9 | 100997090 | 1,02E+08 | 8,92414E-08 | 1,01E+08 | rs436366135 | Intron | TBXT,U6, SFT2D1, MPC1, RPS6KA2, RNASET2, CEP43, CCR6, GPR31, U4, TTLL2, UNC93A, ENSBTAG0000051317, ENSBTAG00000050267, ENSBTAG00000054087, ENSBTAG00000053924 | 1 |
| 9 | 97215264 | 98215264 | 1,3445E-07 | 97715264 | rs383130273 | Intron | PRKN, PACRG, bta-mir-2482 | 1 |
| 10 | 32419298 | 33419298 | 6,12362E-07 | 32919298 | rs379496263 | Intergenic | CDIN1, MEIS2, U4 | 1 |
| 14 | 31639330 | 32639330 | 2,12833E-07 | 32139330 | rs43195259 | Intron | CPA6, PREX2, C14H8orf34 | 1 |
| 25 | 8908535 | 9992804 | 8,96384E-08 | 9408535 | rs380720091 | Intron | U6, ATF7IP6, EMP2, TEKT5, NUBP1, TVP23A, CIITA, DEXI, CLEC16A, SOCS1, RMI2, TNP2, PRM1, PRM2, PRM3, ENSBTAG00000052111 | 117 |
| 25 | 11894597 | 12930197 | 1,7496E-07 | 12394597 | rs109954622 | Intergenic | ERCC4 | 12 |
| 26 | 33908564 | 34908564 | 4,31759E-07 | 34408564 | rs210720477 | Intergenic | HABP2, NRAP, CASP7, PLEKHS1, NHLRC2, DCLRE1A, ADRB1, CCDC186, TDRD1, VWA2, AFAP1L2, bta-mir2285dg, ABLIM1, ENSBTAG00000051735, ENSBTAG00000053724 | 1 |

^1^ QTL location, ^2^ SNP location in the genome, ^3^ Candidate genes located within the identified QTL

^1^ QTL location, ^2^ SNP location in the genome, ^3^ Candidate genes located within the identified QTL
