# Supplementary material for: A Genome-Wide Association Study for Tolerance to Paratuberculosis Identifies Candidate Genes Involved in DNA Packaging, DNA Damage Repair, Innate Immunity, and Pathogen Persistence
Source: Front Immunol. 2022 Apr 6;13:820965. doi: 10.3389/fimmu.2022.820965 (PMC9019162; doi:10.3389/fimmu.2022.820965)
Supplement: Supplementary file 2 [file Table_2.docx]

**Supplementary Table 2.** QTLs surpassing the significance threshold (P < 5 × 10^-7^) for evidence of an association with the case-control study 3.

| **BTA^1^** | **QTL start (bp)** | **QTL end (bp)** | **P-value most significant SNP** | **SNP position^2^** |  | **Annotation** | **Genes in QTL^3^** | **Nº of significant SNPs in QTL** |
| --- | --- | --- | --- | --- | --- | --- | --- | --- |
| 2 | 59865687 | 63604873 | 6,93077E-10 | 60829544 |  |  | THSD7B, U6, CXCR4, MCM6, DARS1, LCT, R3HDM1, UBXN4, ZRANB3, RAB3GAP1, MAP3K19, CCNT2, ACMSD, TMEM163, MGAT5, ENSBTAG00000021755, ENSBTAG000000050570, ENSBTAG00000048332, | 52 |
| 3 | 93238076 | 94238680 | 2,61772E-09 | 93738076 | rs381898247 | Downstream | SCP2, ECHDC2, ZYG11A, COA7, SHISAL2A, GPX7, TUT4, PRPF38A, ORC1, CC2D1B, ZFYVE9, bta-mir-2285bc, ENSBTAG00000054697, ENSBTAG00000052231 | 4 |
| 3 | 1,01E+08 | 1,02E+08 | 6,03571E-10 | 101071736 | rs382504851 | missense | TESK2, MUTYH, TOE1, HPDL, ZSWIM5, UROD, | 1 |
| 3 | 1,12E+08 | 1,13E+08 | 8,28134E-09 | 112113272 | rs381266962 | intron | CSMD2, HMGB4, U6, ZSCAN20, EFHD1, GIGYF2, KCNJ13, SNORC | 2 |
| 5 | 27624006 | 28710231 | 8,59593E-08 | 28210231 | rs381805772 | intergenic | KRT7, KRT80, ATG101, NR4A1, ACVR1B, TAMALIN, SMIM41, ANKRD33, SCN8A, FIGNL2, U6, SLC4A8, GALNT6, CELA1, BIN2, SMAGP, DAZAP2, TFCP2, POU6F1, CSNRP2, LETMD1, ENSBTAG00000052798, ENSBTAG00000054816, ENSBTAG00000054136, ENSBTAG00000016166, ENSBTAG00000049194, ENSBTAG00000049207, ENSBTAG00000023471, ENSBTAG00000051039 | 16 |
| 5 | 87061537 | 88108934 | 1,04173E-08 | 87561537 | rs481379915 | Intron | ETNK1, C2CD5, ST8SIA1, ENSBTAG00000026611 | 22 |
| 15 | 28617881 | 29617881 | 2,90766E-07 | 29117881 | rs380966300 | intergenic | IL10RA, TMPRSS4, SCN4B, SCN2B, JAML, MPZL3, MPZL2, CD3E, CD3D, CD3E, CD3G, UBE4A, U6, ATP5MG, KMT2A, SNORA70, IFT46, TMEM25, ARCN1, bta-mir-2285ak, PHLDB1, TREH, bta-mir-12011, DDX6, 5S_rRNA, CXCR5, BCL9L, UPK2, FOXR1, CENATAC, TRAPPC4, SLC37A4, RPS25, ENSBTAG00000042236 | 1 |
| 16 | 71510469 | 72510839 | 2,26389E-07 | 72010469 | rs210839825 | Intron | INTS7, LPGAT1, NEK2, SLC30A1, RD3, TRAF5, RCOR3, KCNH1, ENSBTAG00000048992 | 2 |
| 21 | 43787957 | 44809342 | 5,37813E-09 | 44287957 |  |  | NPAS3, EGLN3, bta-mir-2285cy, ENSBTAG00000050690, ENSBTAG00000051703 | 2 |
| 23 | 3316206 | 4364138 | 1,13969E-07 | 3816206 | rs136275170 | intergenic | ENSBTAG00000051082, ENSBTAG00000021237, ENSBTAG00000033515, ENSBATAG00000046193 | 5 |
| 25 | 11894597 | 12928660 | 3,609E-07 | 12394597 | rs109954622 | intergenic | ERCC4 | 3 |
| 27 | 1567746 | 3549191 | 1,00502E-08 | 2067746 | rs133482742 | intergenic | CSMD1, U6 | 16 |

^1^ QTL location, ^2^ SNP location in the genome, ^3^ Candidate genes located within the identified QTL
